# Life After Secretion—*Yersinia enterocolitica* Rapidly Toggles Effector Secretion and Can Resume Cell Division in Response to Changing External Conditions

**DOI:** 10.3389/fmicb.2019.02128

**Published:** 2019-09-13

**Authors:** Bailey Milne-Davies, Carlos Helbig, Stephan Wimmi, Dorothy W. C. Cheng, Nicole Paczia, Andreas Diepold

**Affiliations:** Department of Ecophysiology, Max Planck Institute for Terrestrial Microbiology, Marburg, Germany

**Keywords:** bacterial protein secretion, host-pathogen interaction, regulation of virulence mechanisms, protein translocation, *Yersinia enterocolitica*, enteropathogens

## Abstract

Many pathogenic bacteria use the type III secretion system (T3SS) injectisome to manipulate host cells by injecting virulence-promoting effector proteins into the host cytosol. The T3SS is activated upon host cell contact, and its activation is accompanied by an arrest of cell division; hence, many species maintain a T3SS-inactive sibling population to propagate efficiently within the host. The enteric pathogen *Yersinia enterocolitica* utilizes the T3SS to prevent phagocytosis and inhibit inflammatory responses. Unlike other species, almost all *Y. enterocolitica* are T3SS-positive at 37°C, which raises the question, how these bacteria are able to propagate within the host, that is, when and how they stop secretion and restart cell division after a burst of secretion. Using a fast and quantitative *in vitro* secretion assay, we have examined the initiation and termination of type III secretion. We found that effector secretion begins immediately once the activating signal is present, and instantly stops when this signal is removed. Following effector secretion, the bacteria resume division within minutes after being introduced to a non-secreting environment, and the same bacteria are able to re-initiate effector secretion at later time points. Our results indicate that *Y. enterocolitica* use their type III secretion system to promote their individual survival when necessary, and are able to quickly switch their behavior toward replication afterwards, possibly gaining an advantage during infection.

## Introduction

**Figure 1:**
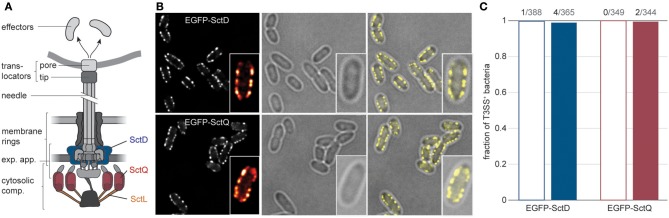
The type III secretion system is expressed in almost all *Y. enterocolitica*, both under secreting and non-secreting conditions. **(A)** Schematic overview of the different substructures of the T3SS (left). Positions of the labeled proteins analyzed in this study are indicated (right). exp. app, export apparatus. **(B)** Micrographs of *Y. enterocolitica* ΔHOPEMTasd expressing EGFP-SctD (top) or EGFP-SctQ (bottom) from their native genetic localization. Three hours after induction of T3SS expression by temperature shift to 37°C under non-secreting conditions, bacteria were subjected to secreting conditions, and imaged. Left, EGFP fluorescence (insets show fluorescence intensity for one enlarged bacterium in ImageJ red-hot coloring scale); center, corresponding phase contrast images; right, overlay. Larger fields of view and images of bacteria under non-secreting conditions are shown in Supplementary Figure 1. **(C)** Fraction of bacteria with standard expression and distribution of T3SS (multiple membrane foci) for the indicated fusion protein, 3 h after induction of expression of the T3SS under non-secreting conditions (empty bars) or secreting conditions (filled bars) *n* = 344–388. Numbers on top indicate the number of bacteria that do not display multiple visible T3SS, and the number of analyzed bacteria. Secreting and non-secreting conditions refer to incubation in medium with addition of 5 mM EGTA or CaCl_2_, respectively.

Bacteria use their type III secretion systems (T3SS) in a variety of ways to survive in different environments. The virulence-associated T3SS^1^ is a membrane-spanning nanosyringe that is used by various Gram-negative bacteria to export effector proteins into the cytoplasm of eukaryotic host cells, allowing them to modify host cell behavior (Cornelis, 2006; Galán, 2009; Pha, 2016). The T3SS protein complex, commonly named the injectisome, is comprised of an extracellular needle, a series of membrane rings embedding an export apparatus, and several cytosolic components (Figure 1A). The needle creates a continuous channel that connects the bacterium to the host cytoplasm with the help of translocator proteins that form a needle tip and a pore within the host cell membrane (Håkansson et al., 1996; Nauth et al., 2018; Park et al., 2018), thus allowing the translocation of effector proteins. The membrane rings anchor the injectisome in the peptidoglycan and the inner and outer membrane, while the export apparatus facilitates the transfer of effectors from the bacterial cytosol through the needle (Kuhlen et al., 2018). The cytosolic components, which shuttle between the cytosol and the injectisome, bind to chaperone-effector complexes and govern the order of secretion (Akeda and Galán, 2005; Lara-Tejero et al., 2011; Diepold et al., 2015). During the assembly and function of the T3SS, three substrate classes are subsequently exported. The early substrates include the needle subunit and a ruler protein that determines the length of the needle. Once the correct needle length is reached, the hydrophilic translocator, classified as intermediate substrate, is exported and assembles a needle tip (Mueller et al., 2005). At this stage, the T3SS is in a standby mode (Enninga and Rosenshine, 2009), and secretion of the late substrates, the pore-forming hydrophobic translocators and the virulence effectors, can be induced, for example by host cell contact.

Pathogens including *Salmonella, Shigella, Pseudomonas*, and *Yersinia* use their T3SS to promote survival and enhance pathogenicity within the host (Büttner, 2012; Deng et al., 2017). In some pathogenic species, such as *Salmonella enterica* and *Pseudomonas aeruginosa*, T3SS are heterogeneously expressed resulting in two subpopulations (Rietsch and Mekalanos, 2006; Sturm et al., 2011; Rundell et al., 2016). The resulting mixed populations can allow bacteria that express the T3SS to promote the survival of their siblings that do not. This is evident for the T3SS encoded by the *Salmonella* pathogenicity island (SPI)-1, where bacteria utilize their T3SS to promote entry into host cells, but also to induce inflammation of the intestinal lumen and remove competition of the intestinal flora (Stecher et al., 2007; Müller et al., 2009; Knodler et al., 2010; Behnsen et al., 2014). The SPI-1-utilizing bacteria display a retarded growth rate, which is a common trait of actively type III-secreting bacteria (Kupferberg and Higuchi, 1958; Mehigh et al., 1989; Fowler and Brubaker, 1994; Sturm et al., 2011). As a result, bacteria that do not express their T3SS outgrow the SPI-1-active population, which can be interpreted as an investment of the SPI-1-active bacteria into increased chances for their genetically identical SPI-1-inactive siblings (Sturm et al., 2011; Diard et al., 2013; Sánchez-Romero and Casadesús, 2018; Weigel and Dersch, 2018).

*Yersinia enterocolitica* is considered a largely extracellular pathogen that uses its T3SS mainly to prevent phagocytosis, inhibit inflammatory responses and promote dissemination (Navarro et al., 2005; Cornelis, 2006; Galán, 2009; Pha, 2016). Once *Yersinia* are exposed to a temperature of 37°C (e.g., after entering a host organism), they start expressing T3SS components (Skurnik et al., 1984; Lambert de Rouvroit et al., 1992). During infection, *Y. enterocolitica* can come into contact with host cells, such as macrophages, dissociate, and possibly establish contact with further host cells. Contact with a host cell activates the secretion of effectors, called Yops (*Yersinia* outer proteins), by the T3SS (Cornelis, 2002). *In vitro*, this activation can be achieved by the chelation of calcium (Ca^2+^) from the extracellular environment. Under these conditions, the bacteria begin exporting their effectors and upregulate effector expression (Cornelis et al., 1987; Wiley et al., 2007; Dewoody et al., 2013). In this study, we used strains based on wild-type *Y. enterocolitica* expressing all virulence effectors (MRS40), as well as on a strain lacking the six main virulence effectors YopH,O,P,E,M,T, as well as the aspartate-beta-semialdehyde dehydrogenase gene (ΔHOPEMTasd), which is consequently avirulent, auxotrophic for the cell wall component diaminopimelic acid, and can be analyzed under safety class 1 conditions.

Prior studies have mainly focused on the activation of the T3SS by host cell contact or Ca^2+^ chelation. However, the post-secretion events like deactivation, reestablishment of bacterial division and the possibility of reactivation of the T3SS are likely to play an equally essential role in promoting bacterial survival and pathogenesis within the host. We therefore used a fast and quantitative *in vitro* secretion assay to examine the initiation and termination of type III secretion in *Y. enterocolitica*, monitored the rate of cell growth and division throughout these steps, and assessed whether previously T3SS-active bacteria can initiate further secretion events. Our results show that activation and deactivation occur immediately in response to changing external conditions, and that after secretion, bacteria transition back to division within short time, while remaining able to reactivate their T3SS.

## Results

### Expression and Assembly of the *Y. enterocolitica* T3SS Is Uniform and Stable Under Different Conditions

Earlier visualizations of T3SS components within *Y. enterocolitica* showed that most bacteria express T3SS, which are localized in a non-random pattern of small patches visible as fluorescent foci at the bacterial surface (Diepold et al., 2010, 2017; Kudryashev et al., 2015). To quantify the fraction of T3SS-positive *Y. enterocolitica*, we analyzed how many bacteria within a population displayed this standard pattern of fluorescence for functional EGFP-labeled versions of a T3SS inner membrane ring protein (SctD), and a cytosolic protein (SctQ) at 37°C. Both under non-secreting conditions (presence of 5 mM Ca^2+^ in the medium) and secreting conditions (chelation of Ca^2+^ by addition of 5 mM EGTA), all or almost all bacteria were T3SS-positive (Figure 1B, Supplementary Figure 1). Even after long-term incubation under secreting conditions for 3 h, the vast majority of bacteria (>98%) were T3SS-positive (Figure 1C).

### Activation Kinetics of the *Y. enterocolitica* T3SS by Ca^2+^ Chelation

**Figure 2:**
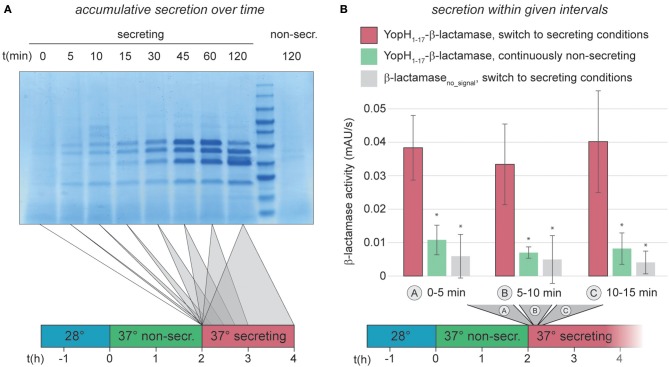
Immediate activation of the T3SS can be measured by an *in vitro* β-lactamase assay. **(A)** Accumulative effector secretion into the culture supernatant during a standard *in vitro* secretion assay using *Y. enterocolitica* MRS40. At the time points indicated (0 min = activation of T3SS secretion by resuspension of bacteria in secreting medium, see also time line at bottom), the culture supernatant of 3 × 10^9^ bacteria was removed and visualized by Coomassie staining of an SDS-PAGE gel. Control (far right), bacteria resuspended in non-secreting medium. **(B)** Quantification of effector export in the indicated time ranges after resuspension of *Y. enterocolitica* ΔHOPEMTasd in secreting medium (see also time line at bottom). Red bars, β-lactamase activity indicative of export of the reporter T3SS substrate YopH_1−17_-β-lactamase; green bars, non-secreting control; gray bars, β-lactamase lacking a T3SS secretion signal under secreting conditions. Error bars indicate standard deviation of the averages of technical triplicates between three biological replicates. ^*^p < 0.05 vs. the YopH_1−17_-β-lactamase, switch to secreting conditions, sample (red bars) in a two-tailed homoscedastic *t*-test. Secreting and non-secreting (non-secr.) conditions refer to incubation in medium with addition of 5 mM EGTA or CaCl_2_, respectively.

The previous results showed that *Y. enterocolitica* populations almost uniformly assemble T3SS injectisomes. We next analyzed the activation and deactivation of these injectisomes in more detail, using an improved version of a previously published enzymatic export assay (Diepold et al., 2015), which measures the export of the reporter construct YopH_1−17_-β-lactamase (Charpentier and Oswald, 2004; Marketon et al., 2005). The updated protocol utilizes the pACYC184 plasmid instead of pBAD, which removes background β-lactamase activity (Supplementary Figure 2A). The higher signal/noise ratio of the modified assay allowed us to reliably quantify secretion in intervals of down to 5 min, and we confirmed that the enzymatic assay itself is not strongly influenced by the used concentration of CaCl_2_ or EGTA (Supplementary Figure 2B). We therefore were able to quantify the initiation of secretion in *Y. enterocolitica* ΔHOPEMTasd within the first 15 min after Ca^2+^ chelation. Within this time range, secretion is difficult to quantify even in an accumulative standard *in vitro* secretion assay (Figure 2A). For the reporter export assay, bacteria were grown at 37°C under non-secreting conditions, allowing for assembly, but not activation of T3SS. Secretion was then activated by resuspension in medium lacking Ca^2+^ and samples were taken every 5 min, and tested for export of the reporter substrate into the supernatant within the following 5 min. The results of the reporter assay clearly show that secretion is fully active at the earliest time range after activation (0–5 min) (Figure 2B), suggesting that effector secretion is initiated immediately by Ca^2+^ chelation. To test whether this fast activation of effector secretion impacts the cellular ATP levels, we determined the adenylate energy charge (([ATP]+0.5[ADP])/([ATP]+[ADP]+[AMP])) of wild-type *Y. enterocolitica* ΔHOPEMTasd, incubated under the same conditions, 10 min after activation of the T3SS. We found that the energy charge of the secreting cultures was not significantly decreased in this time range (Supplementary Figure 3). In line with these findings, the level of secretion of the reporter substrate remained constant throughout the first 2 h after secretion (Figure 2B, Supplementary Figure 4 and data not shown). Secretion activity in a freshly activated wild-type strain was comparable to that of a SctW (YopN) deletion strain, which is calcium-blind and continuously secretes effectors (Supplementary Figure 4), underlining that the level of protein secretion immediately after activation is comparable to the level during ongoing secretion.

### T3SS Effector Secretion Ceases Within Minutes After Removal of the Activating Signal

**Figure 3:**
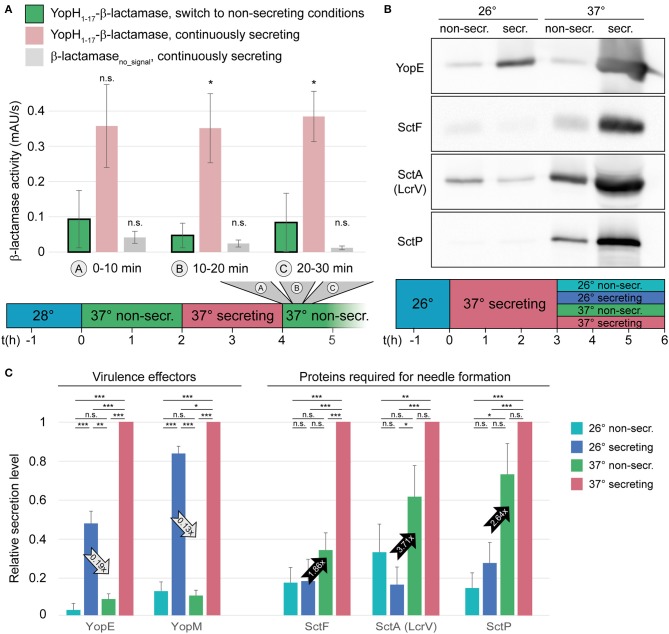
Type III secretion, but not needle formation, is stopped within short time in the absence of activating signal. **(A)** Quantification of effector export in the indicated time ranges after resuspension of *Y. enterocolitica* ΔHOPEMTasd in non-secreting medium (see also time line at bottom). Green bars, β-lactamase activity indicative of export of the reporter T3SS substrate YopH_1−17_-β-lactamase; red bars, secreting control; gray bars, β-lactamase lacking a T3SS secretion signal under secreting conditions. Error bars indicate standard deviation of the averages of technical triplicates between three biological replicates. ^*^p < 0.05 vs. the YopH_1−17_-β-lactamase, switch to non-secreting conditions, sample in a two-tailed homoscedastic *t*-test; n.s., difference not statistically significant. **(B)** The export of different substrate classes is influenced differently by the temperature and the external calcium concentration. Wild-type *Y. enterocolitica* expressing all effectors (MRS40) were grown at 26°C for 1.5 h, and subsequently at 37°C under secreting conditions for 3 h. Afterwards, they were resuspended in different conditions, as indicated (top and time line at bottom) for another 3 h. Proteins secreted by 3 × 10^9^ bacteria were separated on an SDS-PAGE gel and analyzed by immunoblot using antibodies against the indicated proteins, the effector YopE, the needle subunit SctF, the hydrophilic translocator SctA (LcrV), and the ruler protein SctP (*n* = 4, image representative). The respective analysis for bacteria directly subjected to the indicated conditions after incubation at 26°C, Coomassie-based analysis of all secreted proteins, and protein expression controls are displayed in Supplementary Figure 6. **(C)** Relative secretion levels of indicated virulence effectors (left) and proteins required for needle export (right) under the indicated conditions [see time line in **(B)**]. Secretion levels were quantified by densitometric analysis of the bands for the respective proteins in Coomassie-stained SDS-PAGE gels for YopE and YopM (*n* = 3) and immunoblots for YopE (one additional analysis), SctF, SctA (LcrV), and SctP (*n* = 4 in each case), and normalized to the respective secretion level at 37°C under secreting conditions. Error bars display the standard error of the mean; arrows indicate the difference between the influence of the temperature (28°C, secreting conditions) and calcium levels (37°C, non-secreting conditions) and the ratio of secretion under these conditions. Secreting and non-secreting (non-secr.) conditions refer to incubation in medium with addition of 5 mM EGTA or CaCl_2_, respectively. ^*^p < 0.05, ^**^p < 0.01, ^***^p < 0.001 in a two-tailed homoscedastic *t*-test; n.s., difference not statistically significant.

Next, we measured if and how fast the T3SS is inactivated upon reintroduction of Ca^2+^ into the medium. Wild-type *Y. enterocolitica* that had been secreting for 2 h were subjected to non-secreting medium, and the export of the reporter substrate was measured in 10-min intervals afterwards. Already within the first period after Ca^2+^ addition, the export of the reporter strongly dropped compared to the control that was left under secreting conditions (Figure 3A, Supplementary Figure 4). Based on the amount of exported reporter substrate under non-secreting conditions over time (green bars in Figure 3A), deactivation is likely to occur within the first minutes after the removal of the activating signal. Similar to the activation, deactivation of secretion did not increase the energy charge of the bacteria within these time ranges (Supplementary Figure 3).

To further investigate the inactivation of secretion, we aimed to determine, which factor, temperature or Ca^2+^ concentration, has a greater effect on the export of different substrate classes. We therefore analyzed the export of virulence effectors (YopE and YopM), the needle protein SctF, the hydrophilic translocator SctA (= LcrV), and the ruler protein SctP in a wild-type strain expressing all virulence effectors (MRS40). Cultures were grown in secreting conditions and after 3 h, the previously secreting cultures were exposed to non-secreting or secreting conditions, at 26 or 37°C. Our results show that while export of the virulence effectors was strongly repressed by the addition of Ca^2+^, they were secreted in the absence of Ca^2+^, irrespective of the temperature (Figures 3B,C). Export of the needle, translocator and ruler protein, in contrast, was more strongly influenced by the temperature than by the Ca^2+^ level, although this effect was not statistically significant for SctF and SctP (Figures 3B,C). The resuspension steps used in our protocol do not affect assembled needles, and in all cases, cell lysis was negligible; differences in expression levels cannot explain the observed export phenotype (Supplementary Figures 5–7).

### *Y. enterocolitica* Can Resume Growth or Engage in New Secretion Activity After Secretion Has Ended

**Figure 4:**
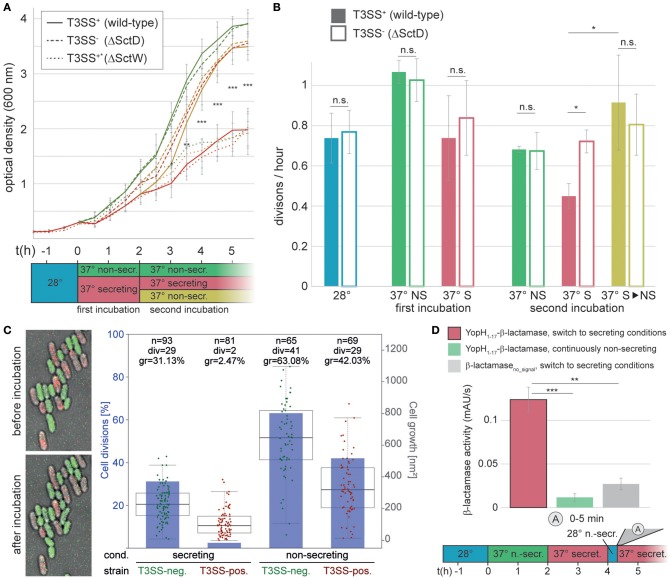
Bacteria can resume division, or engage in another round of secretion after deactivation of secretion. **(A)** Growth curves (optical culture density at 600 nm) of T3SS-positive *Y. enterocolitica* wild-type MRS40 expressing all virulence effectors (T3SS^+^, continuous lines), T3SS-negative bacteria (T3SS^−^, dashed lines), and Ca^2+^-blind constantly secreting bacteria (T3SS^+*^, dotted lines) incubated under the conditions indicated in the time line (bottom). **(B)** Number of bacterial divisions per hour during the different phases, colors as in **(A)**. Filled bars, T3SS-positive bacteria; open bars, T3SS-negative bacteria. Error bars indicate the standard deviation of the averages of technical triplicates between three independent biological replicates. **(C)** Growth and division of T3SS-positive (mCherry-SctL, red) and T3SS-negative (EGFP-SctL ΔSctD, green) *Y. enterocolitica* ΔHOPEMTasd on LB-agarose pads under secreting conditions during the first 2 h of the second incubation period [see **(A)**]. Left, fluorescence micrographs showing growth and division of green (T3SS-negative), but not of red (T3SS-positive) bacteria within the analysis period. Right, quantification of fraction of cell divisions (blue bars and axis on left side) and cell growth (increase in two-dimensional cell area on micrographs) per initial bacterium (box charts and single data points, right axis) for the indicated strains and conditions. Each data point represents a single measurement. The boxes show the median and quartiles (75th and 25th percentile). The whiskers extend 1.5 times the interquartile range until the furthest data point within this range. No standard deviation is displayed. n, number of analyzed bacteria; div, number of bacteria dividing within analysis period; gr, average growth (increase of cell area) within analysis period. Cell growth is statistically significantly different (*p* < 0.001 in a two-tailed homoscedastic *t*-test) for all pairwise comparisons of strains and conditions. **(D)** Quantification of effector export in the indicated time range after resuspension of *Y. enterocolitica* ΔHOPEMTasd in secreting medium after a 15 min incubation in non-secreting medium at 28°C (see time line at bottom). Red bar, β-lactamase activity indicative of export of the reporter T3SS substrate YopH_1−17_-β-lactamase; green bar, non-secreting control; gray bar, β-lactamase lacking a T3SS secretion signal under secreting conditions. Error bars indicate standard deviation of the averages of technical triplicates between three biological replicates. Secreting and non-secreting conditions refer to incubation in medium with addition of 5 mM EGTA or CaCl_2_, respectively. The incubation steps at 28°C (blue bars) are performed in medium with 5 mM CaCl_2_. ^*^p < 0.05; ^**^ *p* < 0.01; ^***^ *p* < 0.001 in a two-tailed homoscedastic *t*-test; n.s., difference not statistically significant. For **(A)**, this statistical analysis applies to the difference between wild-type and ΔSctD under secreting conditions (continuous and dashed red lines), other time points were not statistically significantly different.

Having analyzed the activation and deactivation of type III secretion by external signals, we turned our interest to the events after secretion. Specifically, we wanted to find out whether and when post-secretion *Y. enterocolitica* can resume division and/or re-initiate secretion. To answer the first question, we compared the optical culture density of wild-type *Y. enterocolitica* (MRS40) bacteria that had previously been secreting, and were then either kept under secreting conditions, or subjected to non-secreting conditions. Compared to the non-secreting control, secreting bacteria slow down their division (Figures 4A,B, “first incubation”). Strikingly, previously secreting bacteria that were subjected to non-secreting conditions resumed division within a short time (Figures 4A,B, “second incubation”). This phenotype is linked to T3SS activity, as indicated by the steady division of T3SS-negative ΔSctD bacteria under all tested conditions (Figures 4A,B). As expected, constantly secreting bacteria (the “Ca^2+^ blind” ΔSctW (ΔYopN) strain (Yother and Goguen, 1985), Supplementary Figure 7) displayed growth curves similar to wild-type under secreting conditions, irrespective of the medium. Similar results were obtained on solid medium, where T3SS-positive bacteria did not divide, and only slightly increased their cell volume, under sustained secreting conditions, in contrast to T3SS-negative bacteria (Figure 4C, Supplementary Figure 8). Under non-secreting conditions, both populations displayed a higher rate of growth and division (Figure 4C, Supplementary Figure 8). Taken together, these results indicate that individual *Y. enterocolitica* cells not only can disengage from secretion within a short time, but also quickly resume division in the absence of further stimulating signals.

To determine if *Y. enterocolitica* can also go through repeated cycles of secretion activation and deactivation, we tested the reactivation of secretion in bacteria that had been secreting for 2 h, and where secretion was stopped afterwards by addition of CaCl_2_. These bacteria were incubated in the presence of Ca^2+^ for 15 min at 28°C to suppress the formation of new injectisomes (Figure 3B), and then again subjected to secreting conditions (37°C, absence of Ca^2+^). The secretion of effectors started within the first 5 min after the renewed incubation under secreting conditions (Figure 4D), which shows that type III secretion can be reactivated, and that this occurs within a similarly short time as the initial activation of secretion.

## Discussion

Life after secretion—the deactivation of the T3SS, the recovery of division, and possible additional encounters with host cells—is incompletely understood in *Yersinia*, despite the critical role of these events in the infection process. In this study, we therefore explored the kinetics of activation and, most crucially, deactivation of secretion by external cues, as well as the potential of *Y. enterocolitica* to restart division and to re-initiate secretion afterwards. During infection, when *Y. enterocolitica* enter the Peyer's Patches, bacteria may come into contact with immune cells. In this situation, fast initiation of effector export provides essential defense against phagocytosis and inflammatory signals to the immune system (Grosdent et al., 2002; Navarro et al., 2005; Galán, 2009; Pha, 2016; Philip et al., 2016). Such fast activation of type III secretion has indeed been shown for various bacteria (Enninga et al., 2005; Schlumberger et al., 2005; Mills et al., 2008). Following the interaction with the host, however, *Y. enterocolitica* conceivably benefit from stopping effector export, which may allow them to resume division and disseminate within the host (where the bacteria may again face contact with immune cells). To study T3SS activation and deactivation kinetics in a fast, reproducible and quantitative manner, we used an *in vitro* secretion assay for the reporter substrate β-lactamase in *Y. enterocolitica*, fused to the minimal secretion signal for the native *Y. enterocolitica* virulence effector YopH (Sory et al., 1995). Low and high calcium levels, as used in the assay, have been proposed to mimic the intracellular environment within host cells (low Ca^2+^), and the extracellular host environment (high Ca^2+^), respectively (Fowler and Brubaker, 1994). Our results show that just like activation of secretion (Figure 2), deactivation (Figure 3A) occurs immediately when introduced into secreting or non-secreting media, respectively.

The ability of *Y. enterocolitica* to respond quickly to these external stimuli suggests one or several highly sensitive regulation mechanisms. How exactly host cell contact and changes in the environment, such as the Ca^2+^ levels, are sensed, is still unclear. A number of studies, predominantly performed in *Shigella flexneri* and *Pseudomonas aeruginosa*, suggest that the needle tip senses host cell contact (Veenendaal et al., 2007; Roehrich et al., 2013; Armentrout and Rietsch, 2016), and that the signal is transmitted through rearrangements of the needle subunits to the cytosolic interface of the T3SS (Kenjale et al., 2005; Torruellas et al., 2005; Davis and Mecsas, 2006). Other external factors, such as the Ca^2+^ level and factors inducing other T3SS such as Congo Red might also be sensed at the needle tip and be transmitted the same way. However, the composition of the cytosolic complex of the *Y. enterocolitica* T3SS was shown to be influenced by external Ca^2+^ levels in strains lacking SctD (and therefore also not assembling needles), suggesting a direct sensing mechanism (Diepold et al., 2017). The Ca^2+^-dependent interaction of the ruler SctP and the cytosolic gatekeeper protein SctW in enteropathogenic *E. coli* (EPEC) supports yet another model in which the export of effectors is inhibited as long as a local influx of Ca^2+^ ions occurs through T3SS needles which are not in contact to a host cell (Shaulov et al., 2017).

Notably, in our deactivation experiments, not all substrate classes were affected equally by the Ca^2+^ level. While export of the tested effectors (YopE and YopM) was strongly suppressed upon addition of Ca^2+^, the export of early and intermediary substrates, required for the formation of new needles, continued, albeit at a lower rate. In contrast, a reduction of the temperature to 26°C at continuous low Ca^2+^ levels strongly decreased the export of early and intermediary substrates, but allowed the continued export of the effectors (Figure 3B). This phenotype is most likely not a mere effect of the expression levels of the exported proteins: While the initial expression of all T3SS export substrates is strongly regulated by the temperature, as a consequence of the temperature-dependent expression of the main transcriptional regulator of the T3SS, VirF (Cornelis et al., 1989; Hoe and Goguen, 1993; Böhme et al., 2012), this effect is far less pronounced in a system that has previously been activated by incubation at 37°C under secreting conditions (Supplementary Figures 6A,B). This indicates that in the latter case, mRNA and proteins levels are sufficient for continuous effector secretion at low Ca^2+^ levels, even at 26°C. The secretion of the proteins required for needle formation is more strongly repressed at 26°C (although this effect is only statistically significant for SctA = LcrV), suggesting that the inhibition of the secretion of needle-related proteins and effectors are controlled by additional, and different, regulatory pathways. A similar differential regulation of secretion of the different substrate classes has been shown for *P. aeruginosa* (Cisz et al., 2008) and EPEC (Shaulov et al., 2017), suggesting that this mechanism is conserved amongst different T3SS.

Regulating the events after secretion is particularly important for *Y. enterocolitica*, which differ from other pathogens in that they homogeneously express the T3SS when exposed to 37°C (Figure 1, Supplementary Figure 1), and that the complete population can activate secretion *in vitro* (Wiley et al., 2007). The low fraction of T3SS-negative bacteria even after prolonged *in vitro* secretion (Figure 1C), despite the lower replication rate of these bacteria, highlights the important role of T3SS function during infection in *Y. enterocolitica*. What then happens after a bacterium has survived an encounter with an immune cell? Once effector secretion has ceased, *Y. enterocolitica* benefit from re-initiating faster replication, but have to remain guarded for additional interactions with immune cells. It has been an open question whether re-initiation of replication can be observed *in vitro*, where all injectisomes are activated (Wiley et al., 2007), which might differ from the situation during an infection (Heroven and Dersch, 2014; Avican et al., 2015). We found that *Y. enterocolitica* can quickly resume division when exposed to a non-secreting environment after prior incubation in a secretion-activating environment (Figures 4A–C). This recovery (as well as the cessation of growth and division upon secretion initiation) appears independent of the cellular energy levels, which did not significantly drop under secreting conditions within the tested time range in the used effector-less strain (Supplementary Figure 3). At this point, renewed contact with host cells, especially immune cells, is possible. We have shown that accordingly, previously secreting *Y. enterocolitica* continue to assemble new needles (Figure 3B), and that reactivation of the T3SS is possible and occurs immediately when introduced from non-secreting media back into secreting media (Figure 4D). Reestablishing secretion likely allows the bacteria to defend themselves during future interactions with immune cells. Notably, the experiments to investigate the kinetics of secretion start and stop, as well as the re-initiation of growth and division, were performed in an effector-less strain background (ΔHOPEMTasd). The observed effects are therefore independent of any additional regulatory role of the effectors themselves (Dewoody et al., 2013).

Effector translocation into macrophages prevents phagocytosis and inflammation, which both the interacting bacterium and bystanders benefit from. However, the ability to quickly restart growth after interaction with immune cells implies that *Y. enterocolitica* do not sacrifice to combat immune cells in an altogether altruistic manner, but also attempt to increase individual fitness. Other pathogens deviate from this strategy, and utilize their T3SS differently. In the well-studied example of *Salmonella* Typhimurium SPI-1, only a fraction of bacteria express the T3SS, while the remaining bacteria do not express the T3SS. The SPI-1 activity elicits an inflammatory response that reduces competition with established microflora. Due to the inflammation and the decreased growth rate of the population expressing the SPI-1 T3SS genes, the subpopulation not expressing the SPI-1 genes outgrows both the SPI-1-expressing population and competing bacteria (Sturm et al., 2011; Diard et al., 2013; Weigel and Dersch, 2018). The cooperative virulence of *Salmonella* therefore allows for SPI-1 negative bacteria to colonize the intestinal lumen and thus promotes the competitiveness of the population and efficient invasion of the host (Stecher et al., 2007; Müller et al., 2009; Knodler et al., 2010; Ramos-Morales, 2012; Behnsen et al., 2014).

It is currently unclear whether the suppression of growth during secretion is based on the leakage of ions and amino acids, which might be either co-transported with the secreted proteins or passively diffuse through the T3SS during secretion (Fowler and Brubaker, 1994; Fowler et al., 2009), the metabolic burden caused by biosynthesis, assembly and operation of the T3SS (Brubaker, 2005; Sturm et al., 2011; Wilharm and Heider, 2014; Wang et al., 2016), yet unknown specific regulatory mechanisms, or a combination thereof. Wang et al. showed that in the related *Y. pseudotuberculosis*, activation of the T3SS by Ca^2+^ chelation or host cell contact leads to an increased copy number of the virulence plasmid (Wang et al., 2016). A decrease in virulence plasmid copy number upon deactivation of secretion could therefore increase the amount of energy available for growth and division; however, it is unclear whether such an effect could account for the rather quick recovery of growth and division presented in this study (Figures 4A,C). Notably, on solid medium, cell growth differs between secreting and non-secreting conditions (addition of 5 mM EGTA or CaCl_2_, respectively) even for a T3SS-deficient strain (Figure 4C), which indicates an additional T3SS-independent effect of calcium or other divalent cations on growth on solid medium. Taken together, the finding that growth and cell division are restored within short time after secretion has ceased is most compatible with a direct effect of ion leakage, or a specific regulatory mechanism.

The results of this study describe important parameters of *Y. enterocolitica*'s life after secretion. They show the ability to stop type III secretion and re-initiate growth and division within short time after the loss of the activating signal, while remaining able to enter another round of secretion, and support the notion that *Y. enterocolitica* applies the T3SS in an individual rather than a purely altruistic manner.

## Materials and Methods

Bacterial strains and constructs used in this study are listed in Table 1.

**Table 1:** Bacterial strains and genetic constructs used in this study.

**Strain**	**Relevant characteristics of virulence plasmid**	**References**
***Y. enterocolitica*** **strains**		
MRS40	Wild-type pYV plasmid of *Y. enterocolitica* E40 (pYVe40) ΔblaA	Sory et al., 1995
IML421*asd* (ΔHOPEMTasd)	pYVmrs40 *yopO_Δ2−427_ yopE_21_ yopH_Δ1−352_ yopM_23_ yopP_23_ yopT_135_ Δasd*	Kudryashev et al., 2013
AD4051	pYVmrs40 *ΔsctD*	Diepold et al., 2010
AD4085	pYViml421asd *egfp-sctQ*	Kudryashev et al., 2013
AD4306	pYViml421asd *egfp-sctD*	Diepold et al., 2015
AD4483	pYViml421asd *egfp-sctL ΔsctD*	Diepold et al., 2017
ADTM4521	pYViml421asd *mCherry-sctL*	This study
IM41	pYVmrs40 *sctW_45_* (does not encode *sctW* = *yopN*)	Boland et al., 1996
CH4006	pYViml421asd *sctF_*S*5*C*_*	This study
**Plasmid**	**Relevant characteristics**	**References**
**Plasmids**		
pACYC184	Expression vector	New England Biolabs
pKNG101	oriR6K sacBR^+^ oriTRK2 strAB^+^ (suicide vector)	Kaniga et al., 1991
pAD626	pACYC184::*yopH_1−17_-bla* (β-lactamase from pBAD::His-B cloned in-frame with first 17 amino acids of *Y. enterocolitica* yopH)	This study
pAD627	pACYC184::*bla*	This study
pADTM521	pKNG101 *mCh-sctL* (*pamCherry* and flexible linker cloned in-frame at the N-terminus of *sctL*)	Diepold et al., 2017
pCH06	pKNG101 *sctF_*S*5*C*_* (serine to cysteine point mutation of amino acid 5 in *sctF*)	This study

*Strains and constructs used in this study. Y. enterocolitica strains are based on either the wild-type strain MRS40 or the multi-effector knock-out strain ΔHOPEMTasd (IML421asd), which lacks the virulence effectors YopH, YopO, YopP, YopE, YopM, and YopT, and is auxotrophic for diaminopimelic acid as result from a mutation in the aspartate-β-semialdehyde (asd) gene. The knock-out of the virulence effectors and the asd mutation is used for biosafety purposes, allowing these strains to be handled in a biosafety 1 level environment*.

*E. coli* Top10 was used for cloning and grown on Luria-Bertani (LB) agar plates or in LB medium at 37°C. Chloramphenicol and streptomycin were used to select for expression and suicide vectors at concentrations of 10 and 50 μg/ml, respectively.

*Yersinia enterocolitica* strains were grown over night at 28°C in brain heart infusion (BHI) medium containing nalidixic acid (35 μg/ml), and diaminopimelic acid (60 μg/ml) for ΔHOPEMTasd strains. Day cultures were grown in BHI supplemented with nalidixic acid, diaminopimelic acid where required, MgCl_2_ (20 mM), and glycerol (0.4% v/v). For non-secreting conditions, 5 mM CaCl_2_ was added to the medium, whereas for secreting conditions, residual Ca^2+^ was chelated by addition of 5 mM EGTA.

Plasmids were constructed using Phusion polymerase (New England Biolabs). Mutators for exchange of genes on the pYV virulence plasmid were created as previously described (Diepold et al., 2011). Inserted sequences were confirmed by sequencing (Eurofin Genomics). *Y. enterocolitica* mutants were generated by allelic exchange, resulting in the exchange of wild-type gene sequences with the mutated gene (Kaniga et al., 1991).

### β-Lactamase Assay

ΔHOPEMTasd-based *Y. enterocolitica* were used for the β-lactamase (bla) assays to allow handling in a biological safety level 1 environment. *Y. enterocolitica* harboring pAD626 (pACYC184::YopH_1−17_-bla) or pAD627 (pACYC184::bla) were inoculated from overnight cultures to an optical density at 600 nm (OD_600_) of 0.10 (0.12 in validation experiment) in non-secreting BHI medium. After shaking incubation at 28°C for 1.5 h, the culture was shifted to 37°C to induce the *yop* regulon. After 2 h (1.5 h in the initial validation experiment) of incubation at 37°C, bacteria were collected (unless stated differently, bacteria were collected by centrifugation (2,400 g, 4 min, 37°C), and resuspended in an equal amount of fresh medium pre-warmed to 37°C throughout the protocol), and resuspended in secreting BHI medium. Over the next 15 min, the activation kinetics analysis was performed (see below). To determine the deactivation kinetics, cultures were incubated for 2 h at 37°C in secreting medium and were then collected and resuspended in non-secreting medium. Over the next 30 min, the deactivation kinetics analysis was performed (see below). To determine the reactivation kinetics, cultures were incubated in the secreting medium for 2 h at 37°C and were then collected and resuspended in non-secreting medium pre-warmed to 28°C. Cultures were incubated at 28°C for 15 min, collected and resuspended in secreting medium at 37°C, where the reactivation kinetics analysis was performed.

Samples for the kinetics analysis of activation, deactivation, and reactivation of secretion were treated as follows: after resuspension, 400–800 μl samples were removed from the culture at the indicated time points (0, 5, 10 min for activation; 0, 10, 20 min for deactivation; 0 min for reactivation), collected, resuspended in fresh medium as indicated and incubated in a table top shaking incubator at 37°C, 800 rpm for 5 min (activation and reactivation) or 10 min (deactivation). After this incubation, bacteria were removed by centrifugation (16,000 g, 2 min), and the supernatant was stored at room temperature until all samples of one experiment were collected. For each sample, 100 μl supernatant were added to a Sarstedt TC-Platte 96 well plate in triplicates. 10 μl/well of β-lactamase substrate solution (10 μM Nitrocefin (Merck) in phosphate buffered saline) were added. β-lactamase activity was quantified by the increase in absorbance at 483 nm, caused by β-lactamase catalyzed hydrolysis of Nitrocefin, for 40 rounds of 30 s each at 30°C using a Tecan Infinite 200 Pro photometer. The results are averages of β-lactamase activity, determined by linear regression within the linear range of the absorbance, of three independent experiments that were run in technical triplicates for each experiment.

The initial validation assay was performed as described above with the following modifications: 200 μl supernatant was added to a Sarstedt TC-Platte 96 well plate in triplicates, and 20 μl/well of β-lactamase substrate solution (20 μM Fluorocillin Green 495/525 (Life Technologies in 0.1 M Tris-HCl pH 7.5) was added. β-lactamase activity was quantified by the increase in fluorescence caused by β-lactamase catalyzed hydrolysis of the substrate, measured at 495 ± 5 nm excitation and 525 ± 10 nm emission for 30 rounds every 30 s using a Tecan Infinite 200 Pro photometer. The result is the increase of fluorescence over time of one experiment that was run in a technical triplicate.

### *In vitro* Secretion Time Course

*Yersinia enterocolitica* (MRS40) were selected for the *in vitro* secretion assay to compare the effector export over time. *Y. enterocolitica* were grown as stated above for the β-lactamase assay. At each indicated time point (0, 5, 10, 15, 30, 45, 60, and 120 min secreting; 120 min non-secreting) following activation, 2 ml of culture samples were taken for further analysis (see secretion analysis).

### Growth Curve Experiment

*Yersinia enterocolitica* MRS40, AD4051 (ΔSctD), and IM41 (ΔSctW = YopN) cultures for growth curve experiments were inoculated from overnight cultures to an OD_600_ of 0.12 in non-secreting BHI medium. After incubating at 28°C for 1.5 h, the culture was divided in three parts and collected (2,400 g, 4 min, 37°C). The pellet was then resuspended in either non-secreting medium (one part) or secreting medium (two parts), both pre-warmed at 37°C, to induce the *yop* regulon. The cultures were incubated at 37°C for 2 h, and collected again (2,400 g, 4 min, 37°C). One previously secreting culture was resuspended in secreting medium and the second was resuspended in non-secreting medium; the previously non-secreting culture was resuspended in fresh non-secreting medium, all pre-warmed at 37°C. Cultures were incubated at 37°C for 3.5 h. Throughout the experiment, the optical density at 600 nm wavelength (OD_600_) of all cultures was measured every 30 min in a 1:3 dilution. The number of divisions per time for each culture was determined using the OD_600_ values for −1.5 h and 0 h (28°C), 0 h and 2 h (first incubation), and 2 h and 4 h (second incubation).

### Secretion Analysis

Culture samples were centrifuged (20,000 g, 5 min, 4°C) and 1.8 ml of the supernatant (SN) was collected. SN proteins were precipitated using trichloroacetic acid 10% (w/v) final for 24–48 h at 4°C. Proteins were separated on 4–20% gradient SDS-PAGE gels (BioRad). Samples were normalized to contain the proteins secreted by 0.4 OD units of bacteria (the equivalent of 0.4 ml culture at an OD_600_ of 1). Secreted proteins were stained with “Instant Blue,” Coomassie-based staining solution (Expedeon). Immunoblots were carried out using rabbit polyclonal primary antibodies against *Y. enterocolitica* SctP (MIPA57, 1:3000), YopE (MIPA73, 1:1000), SctA (LcrV) (MIPA220, 1:2000), or SctF (MIPA223, 1:1000), goat anti-rabbit secondary antibodies conjugated to horseradish peroxidase (Dako, 1:5000), and ECL chemiluminescent substrate (Pierce).

### Fluorescence Microscopy—Visualization of Growth Under Secreting and Non-secreting Conditions

*Yersinia enterocolitica* AD4483 (ΔHOPEMTasd EGFP-SctL ΔSctD) (Diepold et al., 2017) and ADTM4521 (ΔHOPEMTasd mCherry-SctL) cultures for microscopy were inoculated from overnight cultures to an OD_600_ of 0.15 in secreting medium. Cultures were incubated at 28°C for 1.5 h and then shifted to 37°C for 2 h. Next, 750 μl of each culture were centrifuged (2,400 g, 3 min), and resuspended in 100 μl of either secreting or non-secreting medium for both strains. 2 μl of resuspended culture were spotted on preheated (37°C) agarose pads containing 1.5% (w/v) low melting agarose, LB, nalidixic acid (35 μg/ml), glycerol (0.4% v/v), MgCl_2_ (20 mM) and either oxalate (20 mM) for secreting conditions, or CaCl_2_ (5 mM) for non-secreting conditions. Bright field images were taken every 5 min and fluorescence images were taken every 20 min for 2 h at 37°C with a Deltavision Spectris optical sectioning microscope (Applied Precision) using a 100x oil immersion objective (Olympus) with a numerical aperture of 1.40. The exposure time was set to 0.2 s, with a light intensity of 32% for bright field, 587 nm and 485 nm excitation lights. Following image acquisition, images were deconvolved using softWoRx 5.5 (standard “conservative” settings). Images were further processed with ImageJ-Fiji (National Institute of Health) using a binary mask for measuring cell growth. Red and green fluorescence was manually adjusted to discriminate T3SS-positive and T3SS-negative bacteria, respectively.

### Fluorescence Microscopy—Quantification of Assembled T3SS Under Secreting and Non-secreting Conditions

ΔHOPEMTasd-based *Y. enterocolitica* AD4085 (EGFP-SctQ) and AD4306 (EGFP-SctD) cultures for microscopy (Kudryashev et al., 2013; Diepold et al., 2015) were inoculated from an overnight culture to an OD_600_ of 0.12. Cultures were incubated at 28°C for 1.5 h. After incubation, cultures were shifted to 37°C for 3 h. Next, 400 μl of culture was centrifuged (2,400 g, 2 min) and concentrated in 200 μl microscopy imaging buffer (100 mM HEPES pH 7.2, 100 mM NaCl, 5 mM ammonium sulfate, 20 mM sodium glutamate, 10 mM MgCl_2_, 5 mM K_2_SO_4_, 0.5% (w/v) casamino acids) containing diaminopimelic acid (60 μg/ml) and CaCl_2_ (5 mM) or EGTA (5 mM) according to the imaging conditions (non-secreting or secreting). To test for possible loss of T3SS during extended secretion, cultures were incubated under secreting or non-secreting conditions at 28°C for 1.5 h, and then shifted to 37°C for 3 h. For visualization, 2 μl of resuspended culture where mounted on a 1.5% (w/v) agar pad casted with the same buffer in a depression slide and visualized in a Deltavision Spectris Optical Sectioning Microscope (Applied Precision), equipped with a UApo N 100x/1.49 oil TIRF UIS2 objective (Olympus), using an Evolve EMCCD Camera (Photometrics). The sample was illuminated for 0.15 s with a 488 laser in a TIRF depth of 3440.0, except for the extended secretion assay, where the exposure time was 0.3 s under non-TIRF conditions. The micrographs where then deconvolved using softWoRx 5.5 (standard “conservative” settings) and further processed for presentation with ImageJ-Fiji. Cells were manually counted in several fields of view.

### Needle Staining Protocol

MRS40-based *Y. enterocolitica* CH4006 (SctF_S5C_) and a MRS40 wild-type control were inoculated from overnight cultures to an OD_600_ of 0.15 in secreting medium. Cultures were incubated at 28°C for 1.5 h and then shifted to 37°C for 2 h. At this point, 500 μl were transferred to a 2 ml tube and washed with minimal medium adjusted for secreting conditions. The cells were concentrated in 100 μl microscopy imaging buffer containing EGTA (5 mM) and CF-633 maleimide dye (Sigma-Aldrich, USA) (5 μM) for 5 min. The cells were then collected and washed with microscopy imaging buffer containing EGTA (5 mM) once or four times, as indicated. Images were acquired as z stacks of 11 images with a stacking of 0.15 μm. The micrographs where then deconvolved using softWoRx 5.5 (standard “conservative” settings) and further processed for presentation with ImageJ-Fiji.

### Energy Charge Determination

Intracellular metabolites were extracted from the total cellular biomass of wildtype ΔHOPEMTasd cultures, used in this experiment for biosafety reasons, based on a sequential quenching–extraction approach. 3 ml culture aliquots were pipetted into 9 ml of 60% (v/v) cold methanol (−60°C). Cells were immediately pelleted by centrifugation (10 min, −10°C, 20,000 x g), and the supernatant was removed. Pellets were stored at −80°C until extracted. Intracellular metabolites were extracted by adding a volume equivalent to 300 μl per 3 ml of a sample at an OD_600_ of 1 of both extraction fluid {50% (v/v) methanol, 50% (v/v) TE buffer [10 mM TRIZMA (pH 7.0), 1 mM EDTA]; −20°C} and chloroform (−20°C) to each cell pellet. The resulting mixture was incubated at 4°C for 2 h on a shaking device (Eppendorf shaker) and centrifuged (10 min, −10°C, 20,000 x g). The upper phase of the two-phase system was filtered (0.22 μm, PTFE, 4 mm diameter, Phenomenex) and stored at −80°C until the polar metabolites were analyzed.

Quantification of nucleotides was performed using a LC-MS/MS. The chromatographic separation was performed on an Agilent Infinity II 1290 HPLC system using a SeQuant ZIC-HILIC column (150 × 2.1 mm; 3.5 μm, 100 Å, Merck, Germany) equipped with a 20 × 2.1 mm guard column of similar specificity (Merck, Germany) at a constant flow rate of 0.4 ml/min with mobile phase A being 20 mM ammonium acetate (Sigma-Aldrich, USA) adjusted to pH 9.2 with ammonium hydroxide (Honeywell, USA) and phase B being acetonitrile (Honeywell, USA).

The injection volume was 3 μl. The mobile phase profile consisted of the following steps and linear gradients: 0–1 min from 20% B to 25% B; 1–4 min from 25 to 35% B; 4–5 min from 35 to 80% B; 5–6 min constant at 80% B; 6–7 min from 80 to 20% B; 7–8 min constant at 20% B. An Agilent 6495 ion funnel mass spectrometer was used in negative mode with an electrospray ionization source and the following conditions: ESI spray voltage 3,500 V, sheath gas 350°C at 11 l/min, nebulizer pressure 20 psig and drying gas 225°C at 14 l/min. Compounds were identified based on their exact mass and retention time compared to standards. Extracted ion chromatograms of the [M-H]- forms were integrated using MassHunter software (Agilent, Santa Clara, CA, USA). Absolute concentrations were calculated based on an external calibration curve.

## Data Availability

All datasets generated for this study are included in the manuscript and/or the Supplementary Files.

## Author Contributions

BM-D and AD conceived, designed experiments, and wrote the paper. BM-D, CH, SW, DC, NP, and AD performed the experiments. BM-D, CH, SW, NP, and AD analyzed the data.

### Conflict of Interest Statement

The authors declare that the research was conducted in the absence of any commercial or financial relationships that could be construed as a potential conflict of interest.

## Footnotes

^1^In this manuscript, T3SS refers to virulence-associated T3SS. The common “Sct” nomenclature (Hueck, 1998; Portaliou et al., 2016) is used for T3SS components, see (Diepold and Wagner, 2014) for species-specific names.
