# Supplementary material for: Life After Secretion—Yersinia enterocolitica Rapidly Toggles Effector Secretion and Can Resume Cell Division in Response to Changing External Conditions
Source: Front Microbiol. 2019 Sep 13;10:2128. doi: 10.3389/fmicb.2019.02128 (PMC6753693; doi:10.3389/fmicb.2019.02128)
Supplement: Supplementary file 1 [file Data_Sheet_1.PDF]

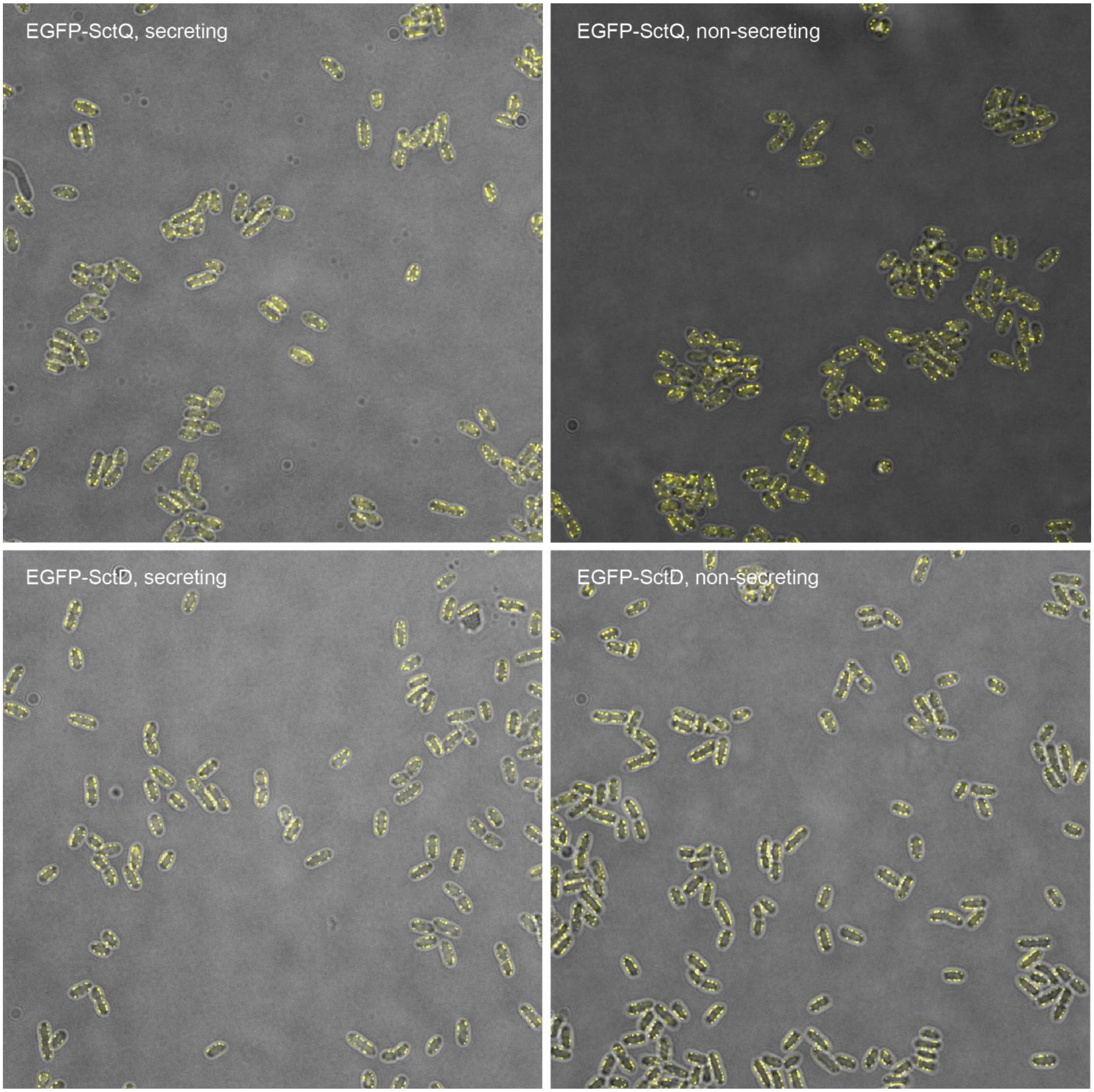

Suppl. Fig. 1 – Expression of labelled T3SS subunits under the indicated conditions

Overlays of phase contrast (grey) and fluorescence images in the green channel (yellow) for *Y. enterocolitica* strains expressing the indicated proteins from their native genetic locus. Bacteria were subjected to the indicated conditions 3 h after induction of T3SS expression by shift to 37°C under non-secreting conditions. Secreting and non-secreting conditions refer to incubation in medium with addition of 5 mM EGTA or $CaCl_2$, respectively.

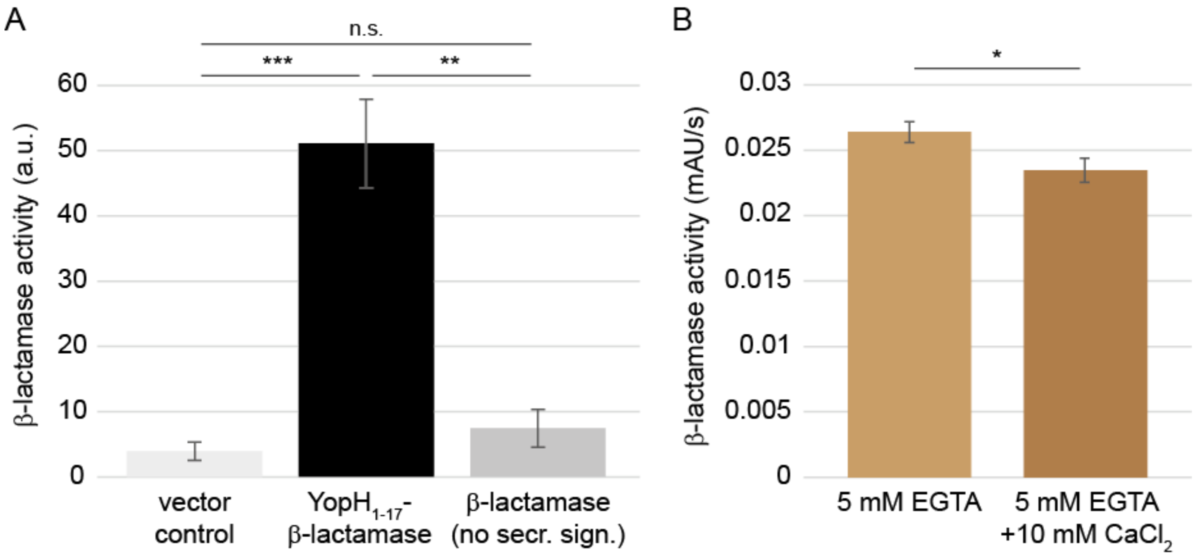

Suppl. Fig. 2 - An improved β-lactamase assay allows the sensitive detection of T3SS-based export of a reporter substrate, YopH$_{1-17}$-β-lactamase

(**A**) Quantification of β-lactamase activity, measured by the increase in Fluorocillin florescence, in the supernatant of strains expressing the indicated proteins for 60 min after resuspension of bacteria in secreting medium. *Y. enterocolitica* ΔHOPEMTasd harboring an empty pACYC184 vector (vector control, light grey), pACYC184::YopH$_{1-17}$-β-lactamase (black), or pACYC184::β-lactamase (no secretion signal, grey). Error bars indicate the standard deviation of a technical triplicate of one experiment. **/***, $p<0.01/0.001$; n.s. difference not statistically significant in a two-tailed homoscedastic t-test. (**B**) Test for direct influence of the Ca^{2+} concentration on the β-lactamase assay. The supernatant of bacteria incubated for 10 minutes after resuspension in secreting medium (containing 5 mM EGTA) was analyzed in a standard β-lactamase assay (see material and methods for details) with or without the addition of 10 mM Ca^{2+}, mimicking the conditions of the β-lactamase assay for non-secreting samples, directly prior to the assay. Error bars indicate the standard deviation of a technical triplicate of one experiment. *, statistical significant difference ($p=0.03$ in a two-tailed homoscedastic t-test).

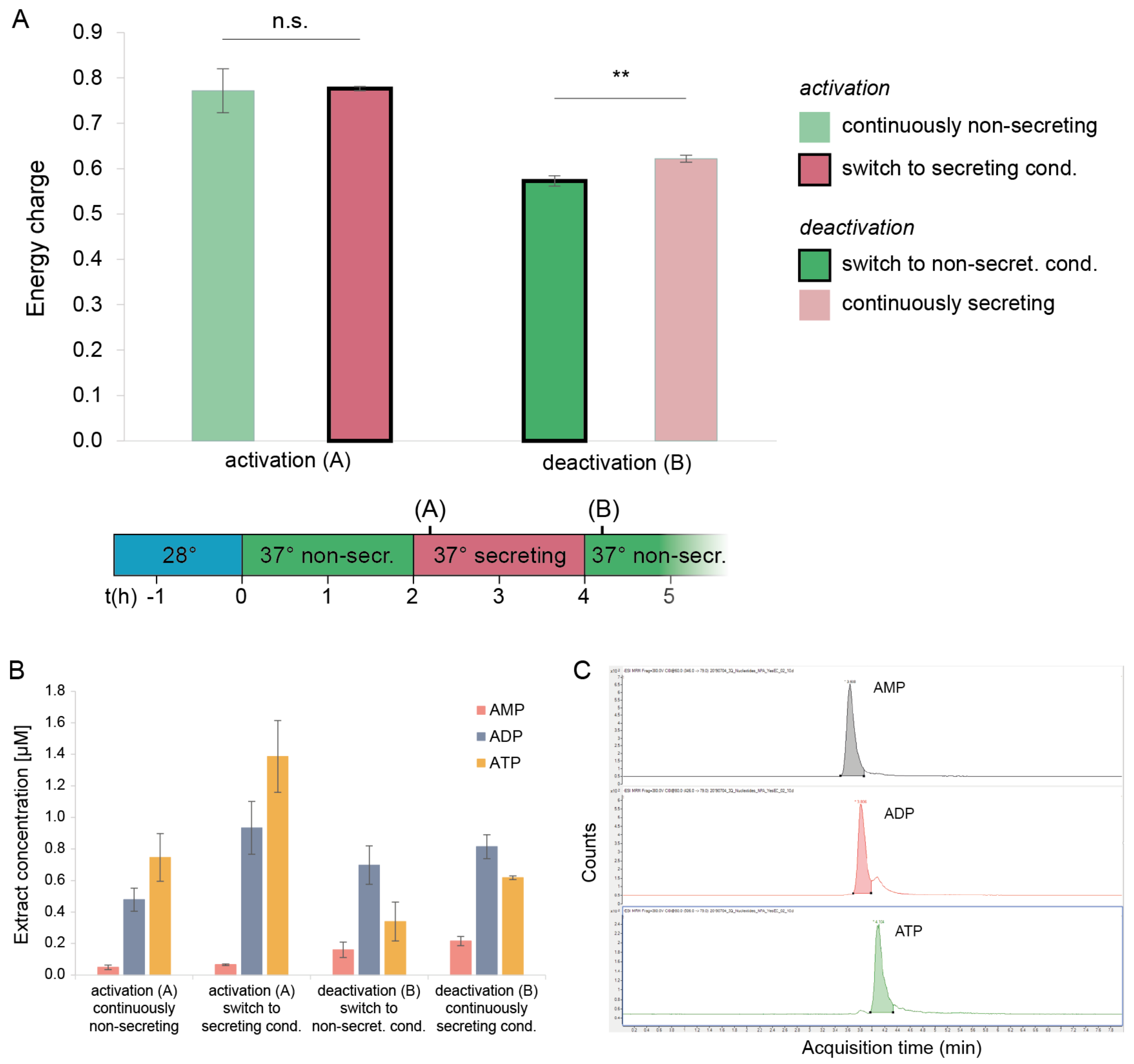

Suppl. Fig. 3 – The ATP energy charge of *Y. enterocolitica* is not increased under non-secreting conditions within the time range used in the activation and deactivation experiments

(**A**) Energy charge, defined as ([ATP]+0.5[ADP])/([ATP]+[ADP]+[AMP]) for activation of secretion (left, relates to Fig. 2B) and deactivation of secretion (right, relates to Fig. 3A). Light green column, continuously non-secreting *Y. enterocolitica* ΔHOPEMTasd; dark red column, switch to secreting conditions; dark green column, switch to non-secreting conditions; light red column, continuously secreting conditions, at the time points indicated in the time line (bottom). Error bars indicate standard deviation of three biological replicates. n.s., no statistical significant difference ($p=0.87$ in a two-tailed homoscedastic t-test); **, statistical significant difference ($p=0.004$). (**B**) Measured AXP concentrations for the experiment shown in (A). (**C**) Chromatographic peak separation of ATP, ADP, and AMP. Secreting and non-secreting (non-secr.) conditions refer to incubation in medium with addition of 5 mM EGTA or $CaCl_2$, respectively.

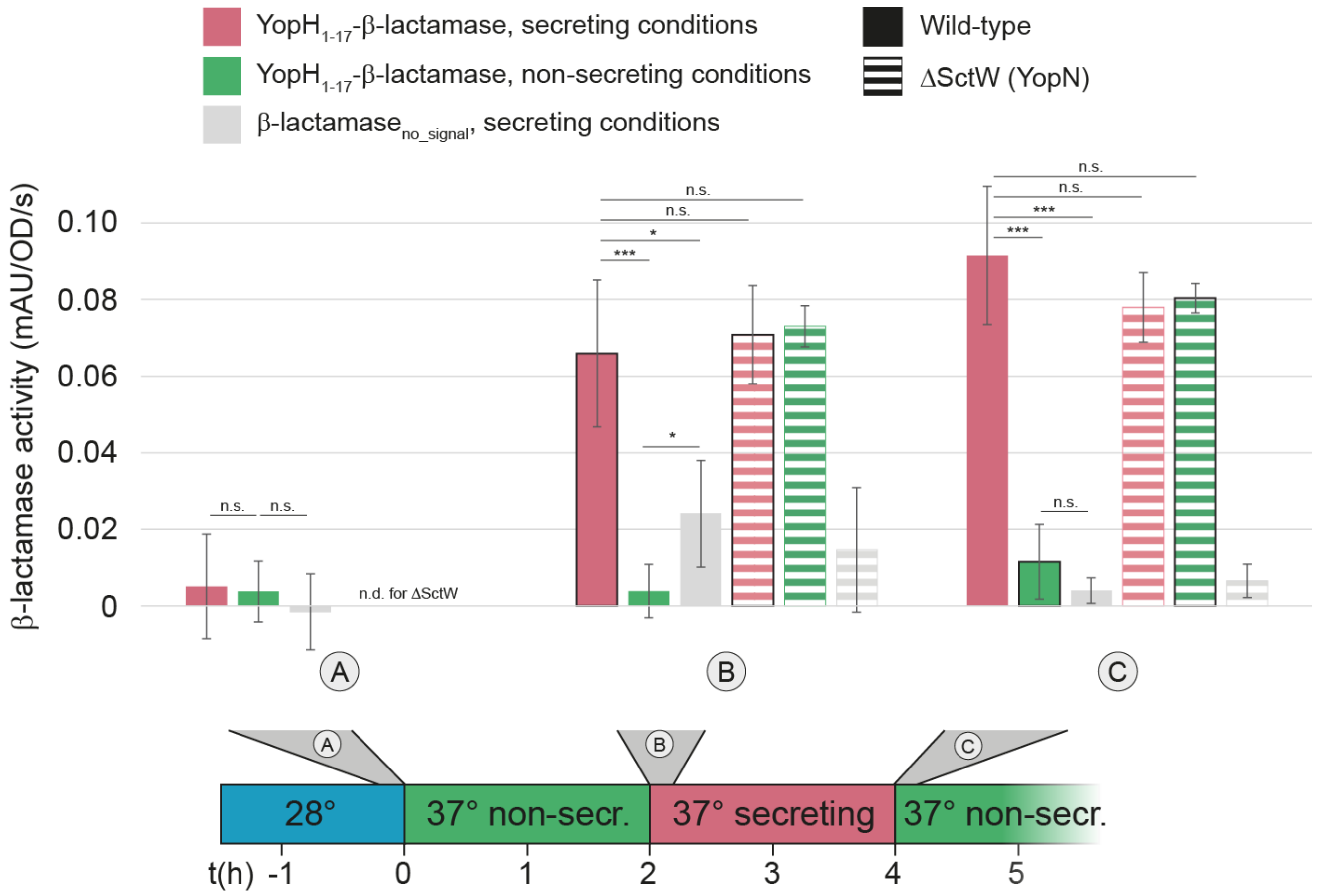

Suppl. Fig. 4 – Comparison of secretion activity at different time points used in this study for wild-type and calcium-blind bacteria.

Quantification of effector export in the indicated ten-minute time ranges (see also time line at bottom) in wild-type (filled) and the calcium-blind ΔSctW (YopN) (striped) strains in a ΔHOPEMTasd strain background. β-lactamase activity, indicative of export of the reporter T3SS substrate $YopH_{1-17}$-β-lactamase, normalized by OD_{600} of culture. Red bars, under secreting conditions; green bars, under non-secreting conditions; grey bars, β-lactamase lacking a T3SS secretion signal under secreting conditions. Black boxes indicate samples shifted into new conditions. Error bars indicate standard deviation of two (ΔSctW strains) to four (wild-type strains) biological replicates. */**/***, p<0.05/0.01/0.001 in a two-tailed homoscedastic t-test; n.s., no statistical significant difference. Secreting and non-secreting conditions refer to incubation in medium with addition of 5 mM EGTA or $CaCl_2$, respectively.

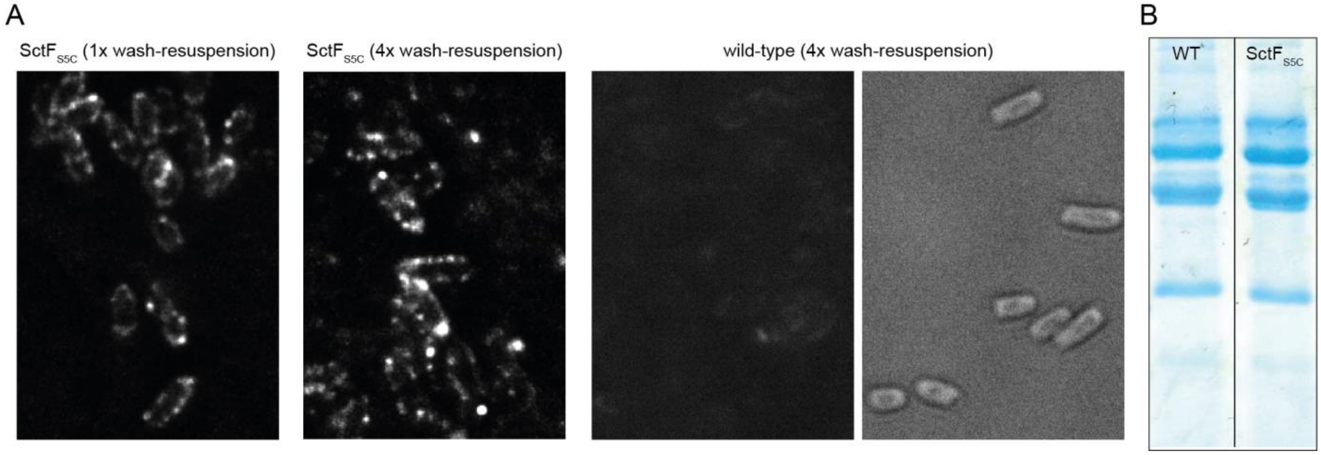

Suppl. Fig. 5: T3SS needles are not significantly affected by gentle resuspension of bacterial cultures

(**A**) Stability of fluorescently marked T3SS needles over several wash-resuspension cycles. To label needles extracellularly, we replaced a serine residue in the unstructured N-terminal region of SctF with a cysteine ($SctF_{S5C}$) in a *Y. enterocolitica* MRS40 wild-type strain. Extracellular SctF was labeled covalently with a fluorescent dye, maleimide-CF633. The amount and intensity of fluorescent foci, representing needles or needle clusters, did not visually differ between strains that were centrifuged and resuspended once (the minimal number for this treatment; left), and four times (center). In contrast, a wild-type strain did not show any clearly discernible foci (right; corresponding transmitted light image on far right for detection of bacteria). (**B**) The strain expressing $SctF_{S5C}$ from the native genetic locus, used in (A), is functional for protein secretion. Coomassie-stained SDS-PAGE gel showing exported proteins (supernatants from $3*10^9$ bacteria) in a wild-type strain and an otherwise identical $SctF_{S5C}$ strain.

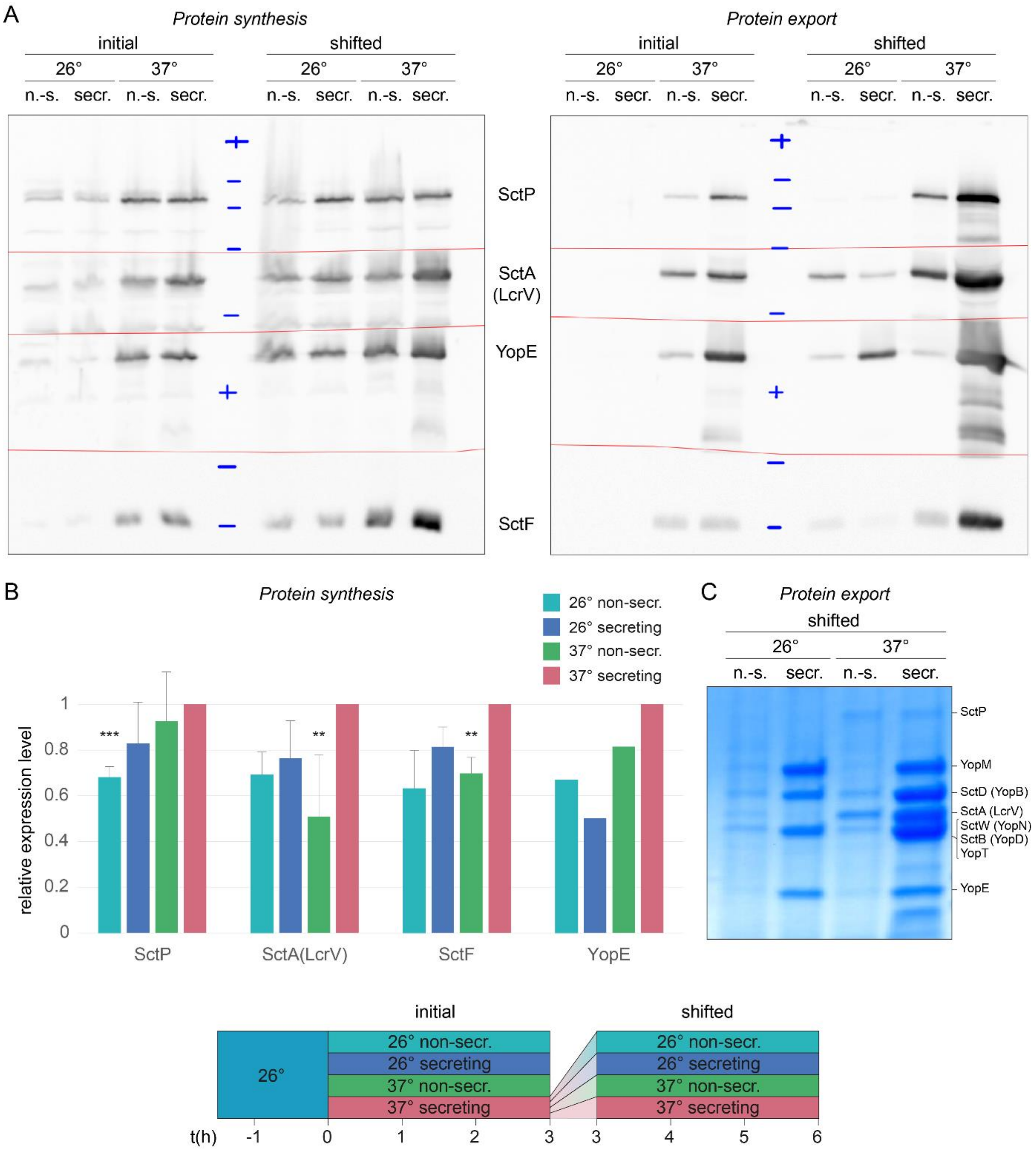

Suppl. Fig. 6 – Protein expression and export by the T3SS in response to different external conditions

(**A**) Immunoblots using antibodies against the indicated proteins to detect protein synthesis (total cell samples from 10^9 bacteria, left) and protein export (supernatants from $3*10^9$ bacteria, right). Initial cultures were subjected to the indicated conditions (n.-s., non-secreting; secr., secreting) directly after 1.5 h at 28°C (0-3 h in the schematic below); shifted cultures were subjected to the indicated conditions after 3 h under secreting conditions at 37°C (3-6 h in the schematic below). Red lines indicate where the membrane was cut to allow incubation with different primary antibodies. Blue crosses indicate position of protein marker (148, 98, 64, 50, 36, 22, 16, 10 kDa from top to bottom).

Expected protein sizes, SctP, 57.7 kDa; SctA (LcrV), 37.3 kDa; YopE, 22.9 kDa; SctF, 9.5 kDa. (**B**) Quantification of protein synthesis for the indicated proteins and conditions (see schematic below). Four independent experiments (one for YopE), performed as in (A), were quantified by densitometry; error bars represent standard errors of the mean. (**C**) Coomassie-stained SDS-PAGE gel of protein export (supernatants from $3*10^9$ bacteria, equivalent to (A)) and tentative assignment of secreted proteins. **/***, $p<0.01/0.001$ in a two-tailed homoscedastic t-test when compared to the respective expression level at 37° C under secreting conditions (red bars). All other combinations show no statistical significant difference.

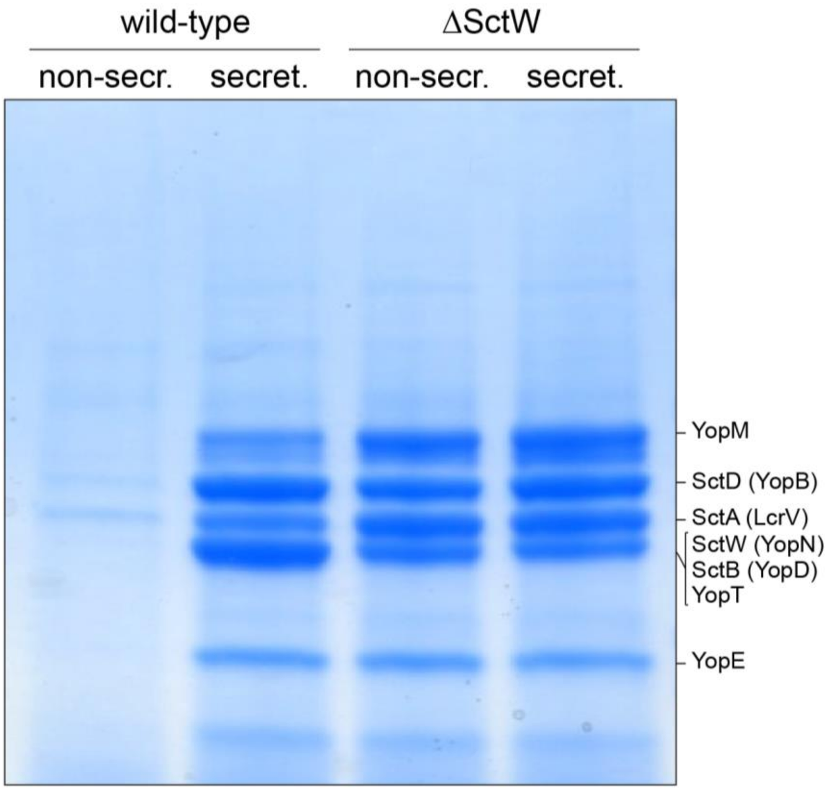

Suppl. Fig. 7: Secretion profiles of wild-type MRS40 and the calcium-blind ΔSctW strain

Coomassie-stained SDS-PAGE gel showing exported proteins (supernatants from $3*10^9$ bacteria) in a wild-type strain expressing all virulence effectors (MRS40) and an otherwise identical ΔSctW (YopN) strain. Right side, tentative assignment of secreted proteins (based on (Diepold *et al*, 2010)). n=3, representative image. Secreting (secret.) and non-secreting (non-secr.) conditions refer to incubation in medium with addition of 5 mM EGTA or $CaCl_2$, respectively.

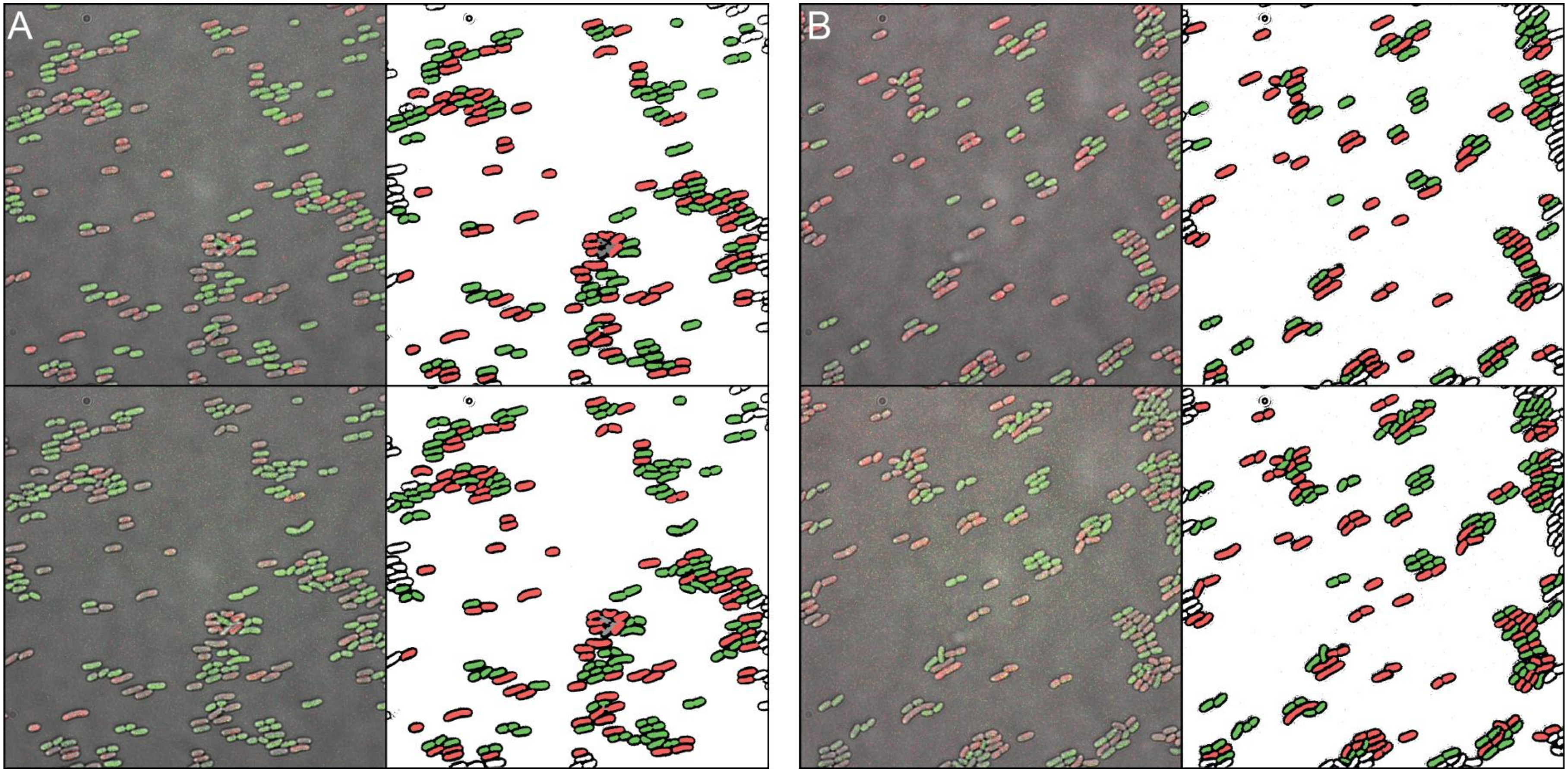

Suppl. Fig. 8 – Growth and division of T3SS-positive and negative bacteria under secreting and non-secreting conditions

T3SS-positive bacteria (ΔHOPEMTasd mCherry-SctL) and T3SS-negative bacteria ((ΔHOPEMTasd EGFP-SctL ΔSctD) from liquid cultures grown under secreting conditions were collected, and mixed in equal ratio, (**A**) under secreting conditions (presence of 5 mM EGTA in the medium), or (**B**) under non-secreting conditions (presence of 5 mM $CaCl_2$ in the medium). The mixed bacteria were immediately spotted onto pre-warmed agarose pads of the respective medium and imaged over time at 37°C. The images displayed were taken immediately after transfer to the agarose pad (top), and two hours afterwards (bottom). Left, overlay of phase contrast images (grey) and fluorescence micrographs (red/green) to distinguish T3SS-positive and negative bacteria. Right, processed images used for strain discrimination and determination of cell growth and divisions.
